# T13. PROGRESSIVE SPONTANEOUS AND SYNCHRONY GAMMA-BAND OSCILLATION DEFICITS IN FIRST EPISODE SCHIZOPHRENIA

**DOI:** 10.1093/schbul/sby016.289

**Published:** 2018-04-01

**Authors:** Yoji Hirano, Naoya Oribe, Toshiaki Onitsuka, Shigenobu Kanba, Martha Shenton, Margaret A Niznikiewicz, Larry J Seidman, Robert W McCarley, Kevin M Spencer

**Affiliations:** 1 Kyushu University; 2 Brigham & Women’s Hospital, Harvard Medical School; 3 Harvard Medical School, Veterans Affairs Boston Healthcare System; 4 Harvard Medical School, Beth Israel Deaconess Medical Center

## Abstract

**Background:**

Deficits in the gamma-band (30–100 Hz) auditory steady-state response (ASSR) and progressive volumetric decreases in the primary auditory cortex have been detected shortly after the onset of schizophrenia (SZ), and may be associated with symptoms such as auditory hallucinations. Disruption of gamma-band oscillation has received considerable interest, as the basic mechanisms underlying these oscillations are understood and are conserved across species. Despite the importance of abnormal gamma-band oscillations in SZ, it remains unclear whether the gamma-band ASSR deficit shows progressive change over time during the early stages of the disease. Moreover, animal models based on NMDA receptor hypofunction demonstrate an increase in spontaneous gamma power, which has been reported in chronic SZ (Hirano et al., JAMA Psychiatry 2015), yet it still remains unclear in first-episode schizophrenia (FESZ). Hence, a longitudinal electroencephalogram study of the spontaneous and synchrony gamma-band oscillation in FESZ is important to better understand the pathophysiology and trajectory of early-stage schizophrenia.

**Methods:**

Subjects were 23 FESZ (14 treated and 9 untreated with antipsychotics), and 39 matched healthy controls (HC). Dipole source localization of dense electrode EEG data was used to examine oscillatory activities in auditory cortices during auditory steady-state stimulation (20/30/40-Hz rates). ICA was used to remove artifacts. Phase locking factor (PLF) and induced power (not phase-locked) were calculated from artifact-free single trial source estimates. Clinical symptoms were assessed by SAPS and SANS. Subjects were recruited as part of the Boston CIDAR Center (www.bostoncidar.org). Test sessions (Time-1/Time-2) were 11.9 months apart.

**Results:**

Compared to HC, FESZ showed reduced 40-Hz ASSR PLF (synchrony gamma) and increased induced gamma power (spontaneous gamma) during continuous auditory stimuli at time-1. Longitudinally, FESZ showed overall progressive reductions in 40-Hz ASSR PLF and progressive increases in induced gamma power, especially within the left auditory cortex. These progressive deficits were not related to antipsychotic medication. Progressive increase of induced gamma power was correlated with increased positive symptoms.

**Discussion:**

We found coincide disruptions of auditory gamma-band oscillation, which showed progressive increase in spontaneous gamma (cortical excitability) and progressive decrease in synchrony gamma (cortical synchrony failure) during continuous auditory stimuli in FESZ. These two apparently distinctive circuit progressive abnormalities already occurred in the very early stage of the disease. We propose that assessing ASSR-PLF and spontaneous gamma in FESZ may provide a sensitive translatable biomarker for the integrity of neural networks that are fundamentally altered in the very early stage of SZ.

